# Associations of Housing Disrepair With Functional Disability Among Older Adult Households in the United States

**DOI:** 10.1093/geroni/igaf122.1777

**Published:** 2025-12-31

**Authors:** Safiyyah Okoye, Karlin Moore, Amruta Patil, Daniel Vader, Craig Pollack, Laura Gitlin

**Affiliations:** Drexel University, Philadelphia, Pennsylvania, United States; Drexel University, Philadelphia, Pennsylvania, United States; Drexel University, Philadelphia, Pennsylvania, United States; Drexel University, Philadelphia, Pennsylvania, United States; Johns Hopkins University, Baltimore, Maryland, United States; Drexel University, Philadelphia, Pennsylvania, United States

## Abstract

Housing disrepair (leaking roofs/walls, broken plumbing/heating) is increasingly common among older adults who often struggle to repair and maintain their homes. Unknown is whether housing disrepair is associated with rates of functional disability and if this association varies for higher and lower income households. Drawing on cross-sectional data (N = 17,610 households with older adults ≥65 years) from the U.S. Census Bureau’s American Housing Survey national survey (2023), we characterized housing disrepair and its associations with functional disability. Housing disrepair was measured as occupant report of at least one of 40 problems across 8 categories (electricity, heating, inside structural, bathroom, kitchen, outside structural, water, sewer). Overall, 36.2% of older adult households reported disrepair; 38.5% among low-income and 34.9% among moderate/high-income households. Functional disability (measured as older adult occupant with difficulty bathing or dressing, doing errands, concentrating or remembering, or climbing or walking stairs) was more prevalent among households with disrepair than without (36.3% vs 24.8%). In a fully adjusted logistic regression model, the odds of an older adult occupant having a functional disability was 1.81 (95% CI 1.65, 1.98) times as high in households with vs without disrepair. In additional adjusted models stratified by income, the odds ratios for the association between disrepair and functional disability were similar in low-income (OR 1.87; 95% CI 1.65, 2.12) and moderate/high-income households (OR 1.77; 95% CI 1.55, 2.02). Further research is needed to establish a causal relationship between housing disrepair, income, and disability and identify causes and interventions to decrease disrepair among older adult households.

